# Supplementary material for: Fine Mapping of qTGW7b, a Minor Effect QTL for Grain Weight in Rice (Oryza sativa L.)
Source: Int J Mol Sci. 2022 Jul 27;23(15):8296. doi: 10.3390/ijms23158296 (PMC9368273; doi:10.3390/ijms23158296)
Supplement: Supplementary file 1 [file ijms-23-08296-s001.zip › ijms-1808014 - Supplemental figures-done.pdf]

Supplemental figures

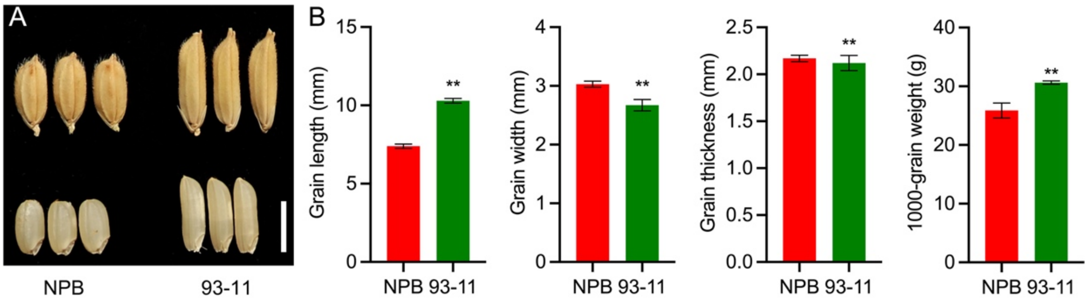

Figure S1. Phenotypic analysis of GL, GW, GT, and TGW in NPB and 93-11. (**A**) Grains with hull and brown grains of NPB and 93-11. (**B**) Statistical analysis of GL, GW, GT, and TGW in NPB and 93-11. Data shown as mean ±SD (*n* = 20). ** $p < 0.01$. Student's t-test was used to generate the *p* values.

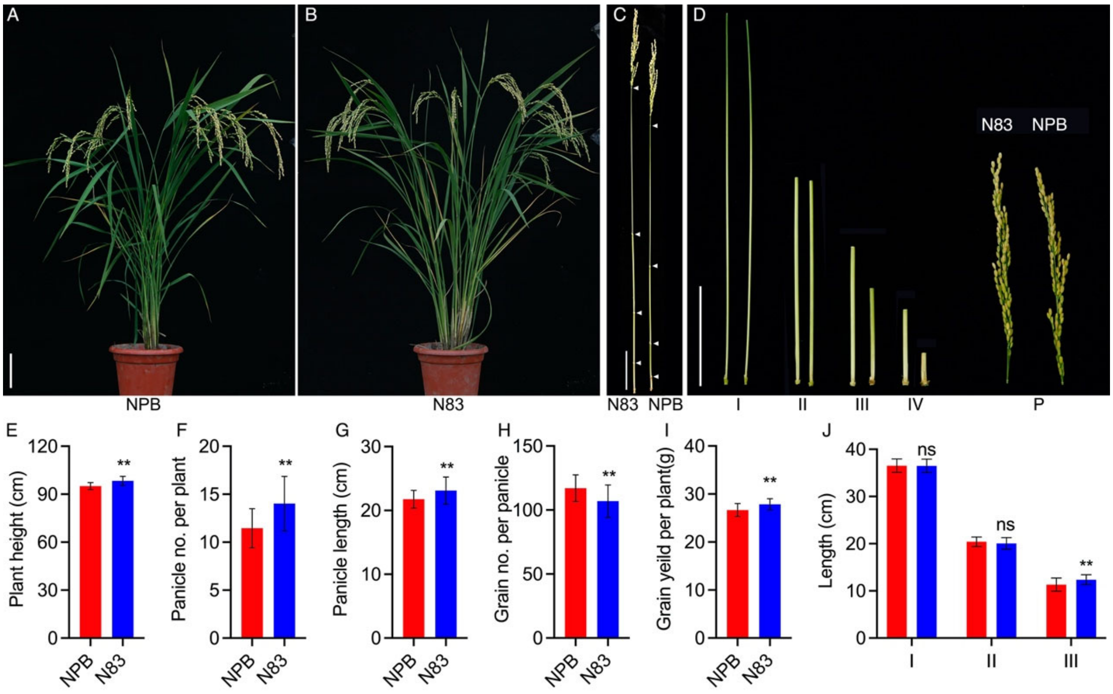

Figure S2. Phenotypic analysis of major agronomic traits of NPB and N83 at maturity. Phenotype of NPB (**A**) and N83 (**B**) plants at maturity. (**C**) Phenotype of the main stem of NPB and N83 plants, where white triangles are marked for each stem node. (**D**) The length of panicle and internode from the main stem of NPB and N83 plants. Scale bar: 10 cm. (**E**–**I**) Statistics of plant type related traits of N83 and NPB, PH (**E**), PN (**F**), PL (**G**), GN (**H**), and GYP (**I**). (**J**) Statistics of the length of the panicle and the last three internodes of NPB and N83. I: 1st internode; II: 2nd internode; III: 3rd internode. Data shown as mean ± SD (*n* = 20). ns, $p > 0.05$, ** $p < 0.01$. Student's t-test was used to generate the *p* values.

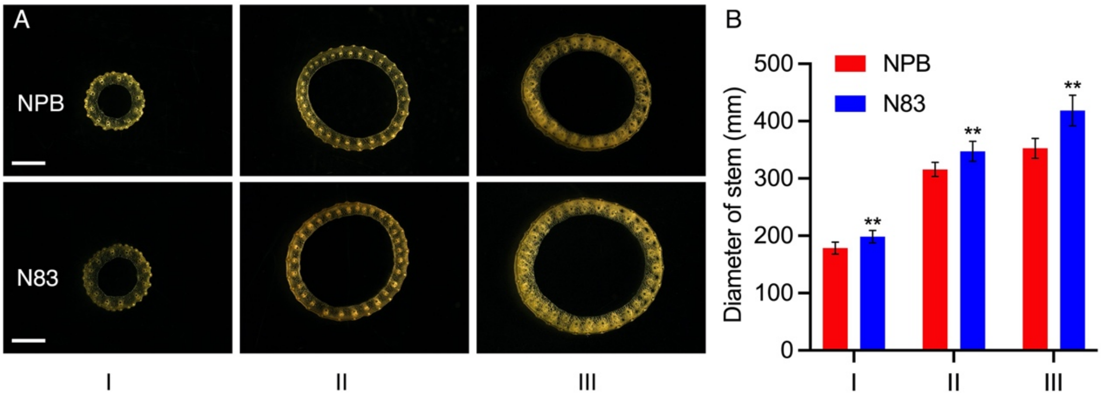

Figure S3. Comparison of the horizontal section in the last tress internodes between NPB and N83. (**A**) Histological observation of the horizontal section of the last tress internodes in NPB and N83. I: 1st internode, II: 2nd internode, III: 3rd internode. Scale bar: 1 mm. (**B**) Statistical analysis of the diameter of the main stem of NPB and N83. Data in (a) (n = 20) are means ± SD, ** $p < 0.01$. Student's t-test was used to generate the p values.

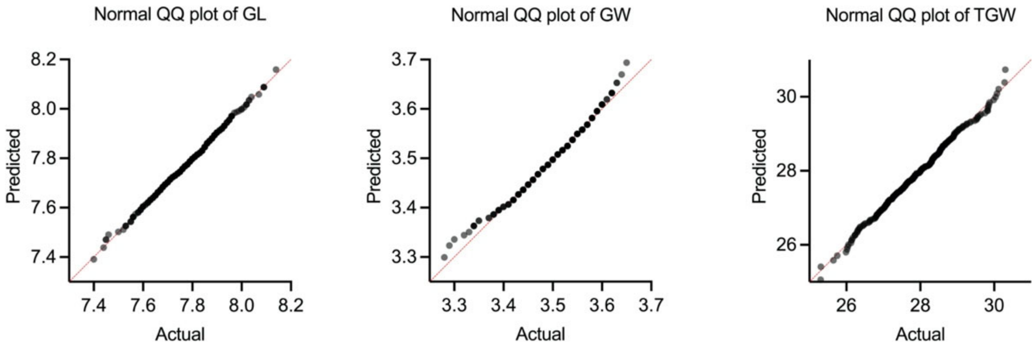

Figure S4. Normality analysis of GL, GW, and TGW of plants in the BC_9F_3 population.
